# Performance of Deep Learning Models in Automatic Measurement of Ellipsoid Zone Area on Baseline Optical Coherence Tomography (OCT) Images From the Rate of Progression of USH2A-Related Retinal Degeneration (RUSH2A) Study

**DOI:** 10.3389/fmed.2022.932498

**Published:** 2022-07-05

**Authors:** Yi-Zhong Wang, David G. Birch

**Affiliations:** ^1^Retina Foundation of the Southwest, Dallas, TX, United States; ^2^Department of Ophthalmology, University of Texas Southwestern Medical Center, Dallas, TX, United States

**Keywords:** deep learning, retinitis pigmentosa, retinal layer segmentation, automatic ellipsoid zone area measurement, outer retinal layer metrics

## Abstract

**Purpose:**

Previously, we have shown the capability of a hybrid deep learning (DL) model that combines a U-Net and a sliding-window (SW) convolutional neural network (CNN) for automatic segmentation of retinal layers from OCT scan images in retinitis pigmentosa (RP). We found that one of the shortcomings of the hybrid model is that it tends to underestimate ellipsoid zone (EZ) width or area, especially when EZ extends toward or beyond the edge of the macula. In this study, we trained the model with additional data which included more OCT scans having extended EZ. We evaluated its performance in automatic measurement of EZ area on SD-OCT volume scans obtained from the participants of the RUSH2A natural history study by comparing the model’s performance to the reading center’s manual grading.

**Materials and Methods:**

De-identified Spectralis high-resolution 9-mm 121-line macular volume scans as well as their EZ area measurements by a reading center were transferred from the management center of the RUSH2A study under the data transfer and processing agreement. A total of 86 baseline volume scans from 86 participants of the RUSH2A study were included to evaluate two hybrid models: the original RP240 model trained on 480 mid-line B-scans from 220 patients with retinitis pigmentosa (RP) and 20 participants with normal vision from a single site, and the new RP340 model trained on a revised RP340 dataset which included RP240 dataset plus an additional 200 mid-line B-scans from another 100 patients with RP. There was no overlap of patients between training and evaluation datasets. EZ and apical RPE in each B-scan image were automatically segmented by the hybrid model. EZ areas were determined by interpolating the discrete 2-dimensional B-scan EZ-RPE layer over the scan area. Dice similarity, correlation, linear regression, and Bland-Altman analyses were conducted to assess the agreement between the EZ areas measured by the hybrid model and by the reading center.

**Results:**

For EZ area > 1 mm^2^, average dice coefficients ± SD between the EZ band segmentations determined by the DL model and the manual grading were 0.835 ± 0.132 and 0.867 ± 0.105 for RP240 and RP340 hybrid models, respectively (*p* < 0.0005; *n* = 51). When compared to the manual grading, correlation coefficients (95% CI) were 0.991 (0.987–0.994) and 0.994 (0.991–0.996) for RP240 and RP340 hybrid models, respectively. Linear regression slopes (95% CI) were 0.918 (0.896–0.940) and 0.995 (0.975–1.014), respectively. Bland-Altman analysis revealed a mean difference ± SD of -0.137 ± 1.131 mm^2^ and 0.082 ± 0.825 mm^2^, respectively.

**Conclusion:**

Additional training data improved the hybrid model’s performance, especially reducing the bias and narrowing the range of the 95% limit of agreement when compared to manual grading. The close agreement of DL models to manual grading suggests that DL may provide effective tools to significantly reduce the burden of reading centers to analyze OCT scan images. In addition to EZ area, our DL models can also provide the measurements of photoreceptor outer segment volume and thickness to further help assess disease progression and to facilitate the study of structure and function relationship in RP.

## Introduction

Recent advances in deep learning (DL) based neural networks have provided new techniques for clinical applications in ophthalmology (2). DL approaches have demonstrated the potential of automatic retinal disease detection and classification from fundus photos and optical coherence tomography (OCT) scan images (3, 4), automatic segmentation of retinal layers and structural features from OCT scan images for quantitative measurements (5–8), and visual function prediction from OCT images (9–12). For instance, deep neural networks have been developed and trained for automatic identification of diabetic retinopathy in retinal fundus photographs (4, 13, 14), for automatic segmentation of retinal layer boundaries in OCT images of dry age-related macular degeneration (AMD) (5), for automated detection and quantification of intraretinal cystoid fluid and subretinal fluid in OCT images of neo-vascular AMD (8), and for predicting glaucomatous visual field damage from OCT optic nerve head *en face* images and retinal nerve fiber layer thickness maps (10).

Automatic analysis of OCT scan images is one of the focus areas of deep learning application in retinal diseases. Efficient and effective techniques for automatic segmentation of retinal layers could significantly reduce the burden of human graders at reading centers to annotate OCT scan images for evaluating disease progression and treatment outcomes. Automatic measurements of retinal layer metrics and structural features can facilitate the study of structure and function relationship and help predict visual function and visual performance with deep learning neural networks. Observation of the structural changes in OCT scan images of various type of retinal diseases suggests that retinitis pigmentosa, an inherited retinal disease, may be one of ideal testing cases for assessing the capability of deep learning approaches for automatic segmentation of retinal layers from OCT scan images.

Retinitis pigmentosa (RP) is a group of genetic eye disorders causing visual impairment. One of the hallmarks of RP is the progressive constriction of central visual field with the advance of the disease. A number of studies using OCT scan images have shown that the structure defects in RP mainly occur in the outer retina as the disease progresses (15–17), with the reduction of ellipsoid zone (EZ) width clearly visible in an OCT B-scan image when EZ transition zone is within the scan area. It has been shown that the loss of EZ band is associated with the loss of visual field sensitivity in RP (18–20). While cystoid macular edema may occur in patients of RP (21) and outer retinal tubulation may form near the end of EZ transition zone (22), the reduction of EZ band width or area remains the primary biomarker of structural changes with the progression of the disease, making EZ a potentially clear target for trained DL models to identify. Previously, image processing–based methods have been employed for automatic segmentation of outer retinal layers in RP (23–25). A more recent study by Loo et al. (26) evaluated a deep learning-based algorithm originally developed for macular telangiectasia (27) for the segmentation of EZ in RP. While they showed that the DL algorithm performed well in segmenting EZ area, it doesn’t provide a measure of other photoreceptor outer segment metrics, such as volume, from OCT volume scans.

In the past 2 years, we have been evolving several deep learning models for automatic retinal layer segmentation in RP and have demonstrated their capability to obtain automatic measurements of outer retinal layer metrics, including EZ width and area, photoreceptor outer segment (OS) length or thickness, area, and volume from SD-OCT images in RP (1, 28, 29). Particularly, we have developed a hybrid model composed of two convolutional neural networks (CNNs) with different architectures, a U-Net (30) for fast semantic segmentation and a sliding-window (SW) CNN model (31, 32) for correcting potential segmentation errors made by the U-Net. With internal testing datasets (i.e., the dataset for model testing was obtained at the same site as that for model training but no overlapping between training and testing datasets), we have shown that the hybrid model consisted of a U-Net and a SW model can be more effective than either model separately for automatic analysis of SD-OCT scan images in RP (1).

However, in previous studies our DL models were mainly evaluated with SD-OCT line B-scan images and have not been tested on real-world external datasets. In addition, one of the shortcomings of the hybrid model is that it tends to underestimate ellipsoid zone (EZ) width and area, especially when EZ transition zone extends to or beyond the edge of the macula (1, 28). We hypothesized that one of the causes for such underestimation may be due to the imbalance in the training data set for the class associated with EZ, either OS area for U-Net or the EZ line for the SW model, since the training dataset naturally included B-scan images with varied EZ width for RP, resulting in decreased (or under) representation of EZ transition zone with the increase of EZ size in the training dataset. In this study, we increased in the training dataset the number of cases of OCT B-scan images having EZ extended to or beyond the macula. U-Net and the SW model were retrained on the new dataset. The performance of the original and new U-Net and hybrid models for automatic measurement of EZ area was then evaluated on an external testing dataset of SD-OCT volume scans obtained from the participants of the Rate of Progression of USH2A-Related Retinal Degeneration (RUSH2A) natural history study by comparing the model’s estimates to that of the reading center’s manual grading. The outcomes of this study would provide us with an insight into the usability and limitations of deep learning approaches in real-world applications of retinal layer segmentation from SD-OCT images in retinitis pigmentosa.

## Materials and Methods

### Deep Learning Models

The deep learning models employed in this study included a U-Net CNN model and a hybrid model that combines U-Net for initial, fast semantic segmentation and a sliding-window (SW) CNN model for refinement. The details of these models have been reported previously (1, 29). All models were implemented in MATLAB (MathWorks, Natick, MA, United States). These models were described briefly as follows.

The U-Net construction followed Ronneberger et al. (30). Specifically, the U-Net consists of a 4-stage encoding (down-sampling) subnetwork to extract features and a 4-stage decoding (up-sampling) subnetwork to achieve semantic segmentation with a bridge component to connect the encoding and decoding stages. The size of input image processed by the U-Net model was 256 × 32 (height × width) pixels. The convolution filter (kernel) size was 5 × 5, and the initial number of feature channels was eight. The “same” padding method (add edges with zeros) is used in convolutional layers so that output image has the same size as the input, which enables the use of a wide range of image sizes. A tile-based approach is employed to segment large images, that is, the U-Net was trained using smaller image patches extracted from larger images. When performing segmentation, a large image is divided into smaller patches for classification, then the classified patches were stitched together to obtain the segmentation of the larger image. In this study, U-Net was trained to classify all pixels in an input image into the following five areas: background, between inner limiting membrane (ILM) and distal (basal) inner nuclear layer (dINL), between dINL and EZ, between EZ and proximal (apical) retinal pigment epithelium (pRPE), and between pRPE and Bruch’s membrane (BM).

The SW model, the second component of the hybrid model, was the same as previously reported (29). This model was based on the framework developed for classifying tiny images (33), and has shown promising results for automatic segmentation of retinal layer boundaries in OCT images of patients with dry AMD (5) as well as patients with RP (29). The SW model included three convolutional layers, three max pooling layers, four rectified linear unit (ReLU) layers, two fully connected layers and a final softmax classification layer. The size of input image handled by the SW model was 33 × 33 pixels. The kernel size was 5 × 5, and the number of initial feature channels was 32. In this study, the SW model was trained to determine if the center pixel of an input image patch was on one of the following retinal layer boundary classes: ILM, dINL, EZ, pRPE, and BM, or was in the background.

The hybrid model was constructed by combining U-Net and the SW model. U-Net was first employed for fast semantic segmentation of OCT B-scan images. Then single-pixel boundary lines were obtained from the semantic segmentation. Specifically, ILM boundary line was defined as the top pixel of the area of ILM-INL; dINL boundary line was defined as the top pixel of dINL-EZ or dINL-pRPE for the parts where EZ was missing; EZ was defined as the top pixel of EZ-RPE; pRPE and BM were defined as the top and bottom pixels of pRPE-BM, respectively. Once five boundary lines were obtained, they were then checked for any line discontinuation or breaks, assuming the actual boundary lines were continuous. The SW model was then employed to re-classify the pixels in the regions surrounding the breaks or gaps to repair any discontinuation along a boundary line. The details of how the hybrid model handles the boundary line breaks and gaps were described in our previous work (1).

### Datasets for the Deep Learning Model Training and Validation

In our previous studies (1, 29), the dataset for training and validation of the DL models was generated from 480 horizontal, 9 mm (30-degree) mid-line B-scan images obtained using a Heidelberg Spectralis (HRA-OCT, Heidelberg Engineering, Heidelberg, Germany) from 20 normal subjects and 220 patients (one scan per eye) with various types of RP who had EZ transition zones visible in the macula. All OCT scans were obtained from a single site. Line B-scans were a mix of SD-OCT high-speed (768 A-scans) or high-resolution (1536 A-scans) B-scans with an automatic real-time tracking (ART) setting of 100. This dataset is now referred as RP240. The Spectralis automatic segmentation of 480 B-scan images in this dataset were manually corrected by one grader using Spectralis software (ver. 1.9.10) for the following five boundary lines: ILM, dINL, EZ, pRPE, and BM.

We have previously shown the capability of the DL models trained with RP240 dataset for automatic segmentation of retinal layers from OCT B-scans (1). However, we found that these models tended to underestimate EZ width or area when EZ extended toward or beyond the edge of the macula (1, 28). We hypothesized that one of possible explanations for this shortcoming was the imbalance of the training dataset because the original RP240 dataset did not include enough cases of EZ transition zone around the edge of the macula and beyond. In this study, we created a revised training dataset which included the original RP240 dataset with an additional 200 B-scans from another 100 patients with RP who had extended EZ area near or beyond the macula. The revised dataset is referred as RP340.

Among the 440 B-scans obtained from 220 participants with RP in the original RP240 training dataset, 31 B-scans had EZ width ≤ 1.0 mm (mean ± SD = 0.79 ± 0.20 mm); 193 had EZ width > 1 mm and ≤ 3.0 mm (1.86 ± 0.56 mm); 145 had EZ width > 3 mm and ≤ 6 mm (4.23 ± 0.79 mm); and 71 had EZ width > 6 mm (7.22 ± 0.69 mm). For the 200 B-scans obtained from 100 participants with RP added to create the new RP340 training dataset, 2 B-scans had EZ width ≤ 1.0 mm (0.78 ± 0.28 mm); 81 had EZ width > 1 mm and ≤ 3.0 mm (2.01 ± 0.55 mm); 70 had EZ width > 3 mm and ≤ 6 mm (4.20 ± 0.80 mm); and 71 had EZ width > 6 mm (7.38 ± 0.78 mm).

For U-Net, the training dataset were image patches of 256 × 32 pixels extracted from B-scan images. The labeling of pixels was based on by their locations. A B-scan image was divided into five areas according to five boundary lines: ILM, dINL, EZ, pRPE, and BM. These areas were labeled as 0, 1, 2, 3, and 4 for background, ILM-dINL, dINL-EZ, EZ-pRPE, and pRPE-BM, respectively. To increase the number of training patches, data augmentation was applied, which included overlapping image patches by 28 pixels horizontally and centering the patches at each boundary line (vertical shift) (1, 28). In this way, a total of 527,488 and 737,024 labeled patches were extracted from RP240 and RP340 for U-Net training and validation, respectively.

For the SW model, the training data were tiny image patches of 33 × 33 pixels extracted from B-scan images. These patches were centered at the pixels on five boundary lines. The labeling of each patch was defined by the class of its center pixel. The pixels on ILM, dINL, EZ, pRPE, or BM boundary lines in a B-scan image were labeled as 1, 2, 3, 4, or 5, respectively. Any pixels in a B-scan image not on these five lines was labeled as 0. The method to generate training dataset for the SW model was described in detail previously (29). A total of 2.88 and 3.98 million classified patches were extracted from RP240 and RP340 datasets for the SW model training and validation, respectively.

All labeled image patches were randomly divided into training set (80%) and validation set (20%). Since the models were trained with small image patches extracted from B-scans, both training and validation datasets contained patches from all participants after patch randomization. The training batch size was 128 patches. Before the training started, all filter weights were set to random numbers. The training stopped after the model was trained for 45 epochs. The initial learning rate was 0.01 for U-Net and 0.05 for the SW model. Learning rate reduced by 10 times every 10 epochs. To accelerate convolutional neural network training and reduce the sensitivity to network initialization (34), a batch normalization layer was inserted between convolutional layers and ReLU layers for the SW model training and between convolutional layers and ReLU layers in the encoding subnetwork for U-Net training. U-Net and the SW model were trained on both RP240 and RP340. The trained models were named as RP240 U-Net, RP240 SW, RP340 U-Net, and RP340 SW. RP240 Hybrid and RP340 Hybrid were the combination of RP240 U-Net and RP240 SW, and RP340 U-Net and RP340 SW, respectively.

Due to the randomization of initial filter weights and stochastic learning algorithms, the models trained on the same dataset may be different each time they are trained, and their performance may have some difference. To evaluation the potential impact of such performance variability on EZ area measurement, all models were trained three times on the same datasets.

### Ellipsoid Zone Area Measurements by the Deep Learning Models

While our deep learning models were trained with mid-line B-scan images, we hypothesized that the models would apply well to most, if not all, of B-scans in a volume scan, given that the models were trained to process small/narrow patches extracted from B-scans. The assumption here is that the image patches used for training the deep learning models would be “building blocks” for all B-scans. The results from our preliminary study provided the evidence to support this hypothesis and showed that the deep learning models trained with mid-line B-scan images can be applied to OCT volume scans for successful segmentation of retinal layers (28).

**FIGURE 1:**
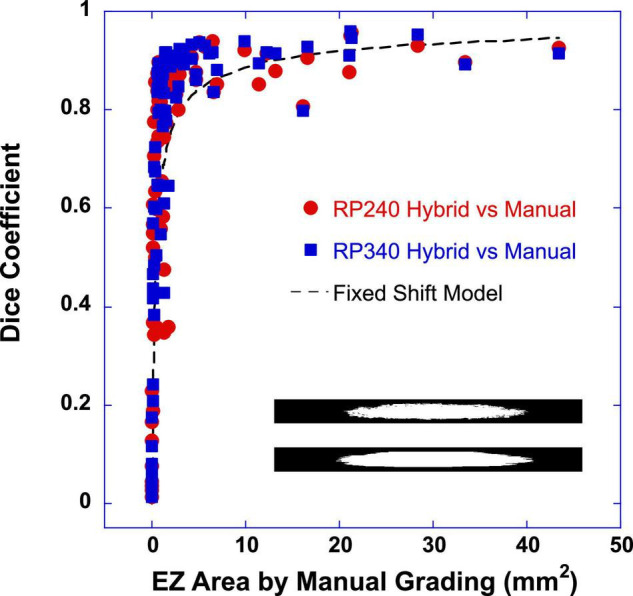
Dice similarity coefficient was plotted as a function of ellipsoid zone (EZ) area determined by the manual grading of the reading center. Close red circles were the dice coefficients between the EZ band area determined by the RP240 hybrid model and that by the manual grading. Closed blue squares were the dice coefficients between the EZ band areas determined by the RP340 hybrid model and the manual grading. The inset images (121 × 1,536 pixels) showed two examples of EZ band segmentations of 121 B-scan lines used for pixel-wise comparison to obtain the dice coefficient (top: by RP340 hybrid model; bottom: by manual grading of the reading center). The dashed line is the output of a simple fixed shift model for dice coefficient between two same size circles. In this example, the constant lateral shift between two circles was 55 pixels (0.315 mm).

**FIGURE 2:**
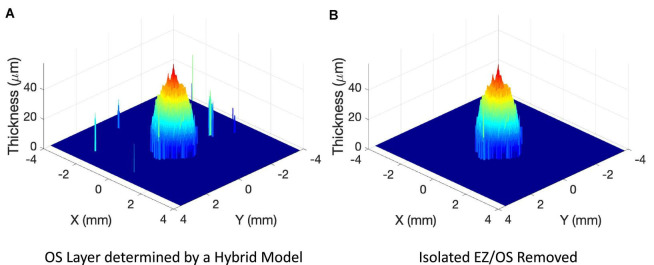
Examples of 3-dimensional plot of photoreceptor outer segment (OS) layer from a 9-mm 121-line SD-OCT volume scan of a patient with retinitis pigmentosa. **(A)** OS layer determined by a hybrid deep learning model described in the method. **(B)** OS layer after off-center isolated local ellipsoid zone (EZ)/OS areas shown in panel **(A)** were removed.

For each B-scan image in a volume scan, ILM, dINL, EZ, pRPE, and BM boundary lines were automatically segmented by the trained DL models. To obtain EZ area measurement, EZ and pRPE in each B-scan image of a volume scan were extracted to obtain photoreceptor outer segment (OS) layer. The EZ band in each line B-scan was marked from the OS layer segmentation to obtain EZ band annotation map of 121 × 1,536 pixels for the pixel-wise comparison with the manual grading (see Figure 1 inset images for examples). The 3-dimensional OS map was reconstructed by interpolating the discrete 2-dimensional OS layers from individual B-scans over the grid of scan area. Figure 2 showed an example of 3-dimensional plot of OS layer from a 9-mm 121-line SD-OCT volume scan of a patient with retinitis pigmentosa. Figure 2A illustrated OS layer determined by a hybrid deep learning model described in the method above, and Figure 2B showed OS layer after off-center isolated small local EZ/OS areas presented in Figure 2A were removed. These isolated off-center local EZ/OS areas were most likely segmentation errors by the DL models. From OS layers such as the one in Figure 2B, EZ area was measured by multiplying the area of a single grid pixel by the number of pixels having measurable OS. Specifically, EZ area was measured by first counting the total number of pixels of measurable OS, then EZ area in mm^2^ was obtained by multiplying total number of pixels by the single pixel area defined as the product of B-scan x-axis resolution and infrared fundus image y-axis resolution in mm/pixel.

Since each model was trained three times separately on the same datasets, three measurements of EZ area were obtained by U-Net and the hybrid model for each OCT volume scan. The mean EZ area measurement was used to compare with that of the reading center. The SW model was not evaluated alone in this study due to the time needed to segment high-density, high-resolution volume scans (1).

### Datasets for the Evaluation of the Deep Learning Models

In this study, we evaluated the performance of the trained U-Net and hybrid models for automatic measurement of EZ area on SD-OCT volume scans of an external dataset obtained from the participants of the Rate of Progression of USH2A-Related Retinal Degeneration (RUSH2A) study (NCT03146078).

RUSH2A is a 4-year natural history study for patients with USH2A mutations, which causes combined vision loss from RP and hearing loss from inner ear dysfunction (35, 36). We have made an agreement with and requested the data from the ongoing Foundation Fighting Blindness (FFB) Consortium RUSH2A study for the evaluation of the DL models trained on the original RP240 dataset as well as on the revised RP340 dataset. Under the data transfer and processing agreement, we received de-identified baseline Spectralis high-resolution 9-mm 121-line macular volume scans as well as their EZ area measurements by a reading center. In this study, a total of 86 baseline volume scans from 86 non-European participants^1^ of the RUSH2A study were included to evaluate the performance of the trained DL models. There was no overlap of patients between the training and the evaluation datasets. No pre-processing was conducted on the received OCT images of RUSH2A data before applying RP240 and RP340 models.

### Data Analysis

The performance of U-Net and the hybrid model to measure the EZ area from the volume scans of the RUSH2A baseline data was evaluated by comparing the model’s results to that of the reading center. Sørensen–Dice similarity, Pearson correlation, linear regression, and Bland-Altman analyses were conducted to assess the agreement and difference between automatic measurements of EZ area by the DL models and the manual grading by the reading center.

## Results

### Dice Similarity Coefficient Between Ellipsoid Zone Band Segmentations by the Deep Learning Models and the Reading Center

The similarity between the DL models and the manual grading of the reading center to determine EZ areas was first evaluated with Sørensen–Dice similarity analysis. Dice similarity coefficient (DSC) was computed between the EZ band segmentation determined by the models and the EZ band annotation by the manual grading of the reading center for the 121 B-scan lines in each volume scan. Figure 1 plots DSC as a function of EZ area of the manual grading. Closed red circles were the DSC between the EZ band segmentations by the RP240 hybrid model and the manual grading. Closed blue squares were the DSC between the EZ band segmentations by the RP340 hybrid model and the manual grading. The inset images (121 × 1536 pixels) in Figure 1 showed two examples of EZ band annotation of 121 B-scan lines used for pixel-wise comparison to obtain the dice coefficient.

It is evident from Figure 1 that when EZ area was very small (<1 mm^2^), dice coefficient varied significantly, ranged from 0.013 to 0.895. The smaller the EZ, the smaller the dice coefficient. When EZ ≥ 1 mm^2^, dice coefficient appeared to be much less varied and tended to reach a plateau. This pattern of DSC changes with the increase of EZ area resembles the behavior predicted by a simple fixed shift model for DSC between two same-size circles, as illustrated by the dashed line in Figure 1. Here the dashed line is the output of the fixed shift model of DSC with a constant lateral shift of 55 pixels (0.315 mm) between two circles.

For easy description, we defined EZ areas into four sub-groups based on the scales of ETDRS macular grid: (1) very small size EZ with the area less than 1 mm^2^ (roughly corresponding to the central subfield of the ETDRS grid); (2) small-size EZ as ≥ 1 mm^2^ and < 7 mm^2^ (inner ring of the ETDRS grid); (3) medium-size EZ as ≥ 7 mm^2^ and < 30 mm^2^ (outer ring of the ETDRS grid) and large-size EZ as ≥ 30 mm^2^ (beyond the ETDRS grid). For EZ area ≥ 1 mm^2^, the mean DSC ± SD between the EZ band segmentations determined by the DL model and the manual grading were 0.835 ± 0.132 and 0.867 ± 0.105 for RP240 and RP 340 hybrid models, respectively. Paired t-test revealed that the 0.032 DSC improvement of RP340 over RP240 was significant (*p* < 0.0005; *n* = 51). For small size EZ (≥ 1 mm^2^ to < 7 mm^2^), the mean DSC ± SD were 0.814 ± 0.145 and 0.851 ± 0.101 for RP240 and RP 340 hybrid models, respectively (*p* = 0.0016; *n* = 38). For medium-size EZ (≥7 mm^2^ to <30 mm^2^), the mean DSC ± SD were 0.895 ± 0.047 and 0.913 ± 0.045 for RP240 and RP 340 hybrid models, respectively (*p* = 0.0072; *n* = 11). For large size EZ (*n* = 2), the sample size was too small to compare RP340 and RP240 models to determine the impact of the re-trained model on the area estimation of large-size EZ. For very small size EZ, there was no significant difference of DSC between RP340 and RP240 models. There was also no difference of DSC between U-Net and the hybrid model.

### Correlation and Linear Regression Between Ellipsoid Zone Areas Measured by the Deep Learning Models and by the Reading Center

**FIGURE 3:**
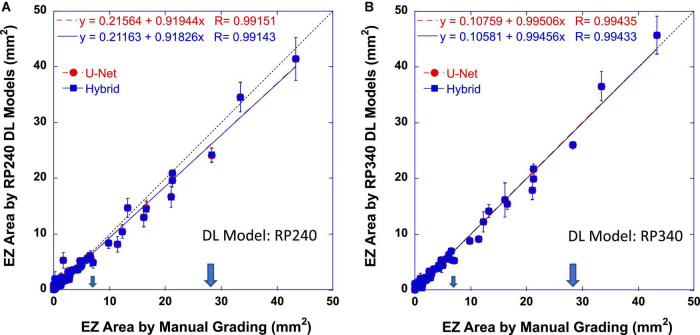
Ellipsoid zone (EZ) areas determined automatically by RP240 **(A)** and RP340 **(B)** deep learning (DL) models as functions of that by the reading center. Red circles and dashed lines are the measurements of the U-Net model. Blue squares and solid lines are the measurements of the hybrid model. Dotted lines have a slope of one. The large arrows mark the central retinal area with a radius of 3 mm from the fovea (28.3 mm^2^). The small arrows mark the central retinal area with a radius of 1.5 mm from the fovea (7.0 mm^2^). Error bars indicate ±1 standard deviation of three measurements by the same model type but trained three times separately on the same datasets. The equations in the plots were the Pearson correlation coefficients (R) and the linear regression fitting result of the data (red for U-Net and blue for the hybrid model).

Dice coefficient assessed the pixel-wise similarity of EZ band annotation of 121 B-scan lines. The similarity between the DL models and the reading center to determine the size of EZ areas in mm^2^ was also evaluated with Pearson correlation analysis and linear regression. Figure 3 plots EZ areas measured automatically by the DL models trained on RP240 dataset (Figure 3A) and on RP340 dataset (Figure 3B) vs. that determined by the manual grading of the reading center. Red circles and dashed lines are the measurements of the U-Net model. Blue squares and solid lines are the measurements of the hybrid model. Dotted lines have a slope of one, indicating perfect agreement for the data points falling on them. The larger arrows mark the central retinal area with a radius of 3 mm from the fovea (28.3 mm^2^). The smaller arrows mark the central retinal area with a radius of 1.5 mm from the fovea (7.0 mm^2^). Error bars indicate ±1 standard deviation of three measurements by the same type of model but trained three times separately on the same datasets. The equations in the plots were the linear regression fitting result and the correlation coefficients (*R*) of the data (red for U-Net and blue for the hybrid model).

Table 1 summaries the results of correlation coefficients and linear regression slopes as well as their 95% confidence intervals (95% CI) for all DL models evaluated in this study. In general, the results showed that EZ area determined by the DL models was highly correlated with that by the reading center (r > 0.99 for both U-Net and the hybrid model). The linear regression analysis showed that automatic measurements of EZ area by the DL models were in close agreement with the manual grading of the reading center. The slope of linear fitting for U-Net and the hybrid model was 0.9 or higher, approaching to one (perfect agreement) for RP340 models. Also listed in Table 1 were coefficients of determination *R*^2^, which were larger than 0.98, suggesting that the agreement between the DL models and the manual grading was 98% or higher.

**TABLE 1:** Summary of correlation coefficients, coefficients of determination (*R*^2^), linear regression slopes, mean differences, as well as mean absolute errors between ellipsoid zone (EZ) areas determined by the deep learning models and that of the reading center (human grading).

EZ area	Correlation coefficient *r* (95% CI)	*R* ^2^	Linear regression slope (95% CI)	Mean difference ± SD (mm^2^)	Absolute error (mean ± SD, mm^2^)
RP240 U-Net vs. Manual Grading	0.991 (0.987–0.994)	0.983	0.919 (0.898–0.941)	−0.129 ± 1.124	0.658 ± 0.917
RP240 Hybrid vs. Manual Grading	0.991 (0.987–0.994)	0.983	0.918 (0.896–0.940)	−0.137 ± 1.131	0.663 ± 0.924
RP340 U-Net vs. Manual Grading	0.994 (0.991–0.996)	0.989	0.995 (0.976–1.014)	−0.087 ± 0.824	0.517 ± 0.645
RP340 Hybrid vs. Manual Grading	0.994 (0.991–0.996)	0.989	0.995 (0.975–1.014)	−0.082 ± 0.825	0.517 ± 0.645

*CI, confidence interval; SD, standard deviation.*

When comparing U-Net and the hybrid model, examination of the individual correlation coefficients in Table 1 reveled that U-Net and the hybrid model trained on the same dataset had the same coefficient. While there might be a small difference in coefficients for RP340 and RP240 models, such small difference was not significant since there was an overlap of 95% CI for correlation coefficient between RP340 and RP240 models vs. manual grading.

On the other hand, while the linear regression slope was almost identical for U-Net and the hybrid models trained on the same dataset as shown in Table 1, the 95% CI of the slopes of RP340 models included one (i.e., not significantly different from a perfect agreement) while that of RP240 models didn’t. Furthermore, there was no overlap of 95% CI for the slope between RP340 and RP240 models, suggesting that additional training data added in RP340 significantly improved the agreement between the DL models and the manual grading for the measurement of EZ areas.

### Bland-Altman Plots—Limit of Agreement

**FIGURE 4:**
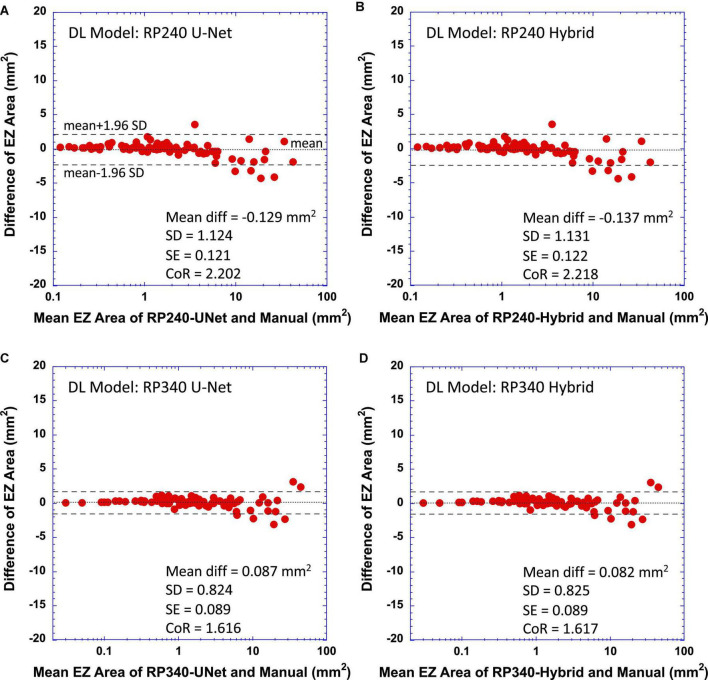
Bland-Altman plots of difference of measurements between deep learning (DL) models and the reading center vs. their mean. **(A)** RP240 U-Net vs. manual grading; **(B)** RP240 hybrid model vs. manual grading; **(C)** RP340 U-Net vs. manual grading; and **(D)** RP340 hybrid model vs. manual grading. Coefficient of repeatability (CoR) is defined as 1.96 times the standard deviation of the difference. Dashed horizontal lines represent ±95% limit of agreement (mean ± CoR). Dotted horizontal lines represent the mean difference.

To further evaluate the performance of the trained DL models, we examined the difference between EZ areas measured by the models and that by the reading center. Figure 4 shows Bland-Altman plots comparing the EZ areas determined by the DL models to that by manual grading of the reading center (Figure 4A for RP240 U-Net vs. manual grading, Figure 4B for RP240 Hybrid, Figure 4C for RP340 U-Net, and Figure 4D for RP340 Hybrid). In each plot, horizontal axis is the mean EZ areas estimated by the DL model and the manual grading, while the vertical axis is the difference of EZ areas by the DL model and the manual grading. The text in each plot lists the values of mean difference (Mean diff), standard deviation of the mean difference (SD), standard error of the mean difference (SE), and coefficient of repeatability (CoR). Dotted horizontal lines indicate the mean difference, and dashed horizontal lines represent ±95% limit of agreement (mean ± 1.96 * SD of the difference). For easy visualization of the data points of smaller EZ areas, the horizontal axes of the plots in Figure 4 are in log scale.

Figures 4A,B showed that, when compared to the manual grading, the original RP240 U-Net and the hybrid models tended to somewhat overestimate small-size EZ area (positive difference) but underestimate medium-size EZ (negative difference). The negative difference of EZ area measurements by the model appeared to become larger when the EZ transition zone was approaching to or around the edge of the macula (3 mm radius from the fovea, corresponding to an EZ area of 28.3 mm^2^).

For the RP340 models, Figures 4C,D showed some reduction of positive biases for small-size EZ and the reduction of negative biases for medium-size EZ when compared to RP240 models (Figures 4A,B). As anticipated, the combined effect of the reduction of negative difference for medium-size EZ and the reduction of positive difference for small-size EZ by the RP340 models when compared to RP240 models improved CoR of the RP340 models to 1.6 mm^2^ from 2.2 mm^2^ of the RP240 models, resulting in the RP340 models having closer agreement with the manual grading. The RP340 models still had a slight bias in EZ area measurement when compared to the manual grading. However, this bias was trivial since 95% CI of standard error of the mean difference included zero. As shown in Figure 4, Bland-Altman analysis revealed a mean difference ± SD of -0.137 ± 1.131 mm^2^ and 0.082 ± 0.825 mm^2^ for RP240 and RP340 hybrid models, respectively. Mean differences ± SD, as well as mean absolute error ± SD, were also reported in Table 1.

Since there were only two cases of large-size EZ, the results did not provide sufficient evidence to determine the impact of re-trained model on the area estimation of large-size EZ.

### Examples of Ellipsoid Zone Areas Determined by the Deep Learning Models

**FIGURE 5:**
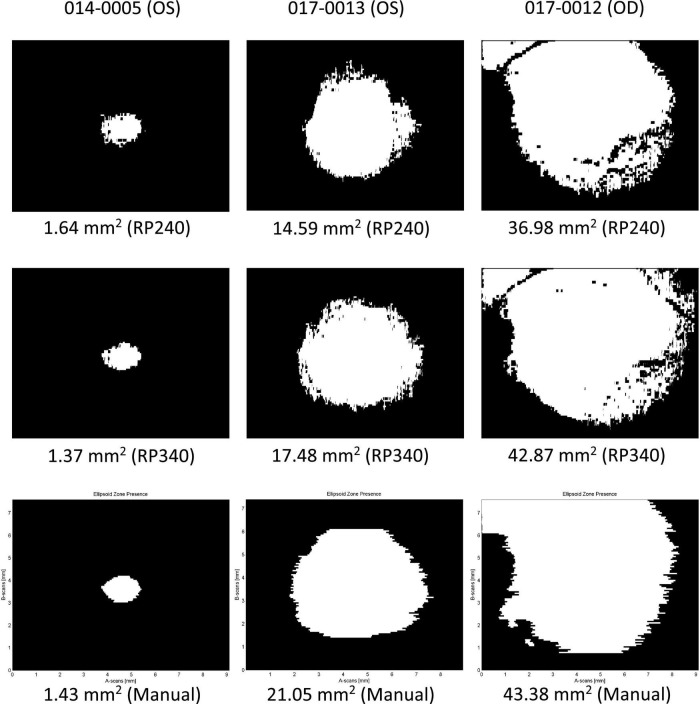
Examples of ellipsoid zone (EZ) area presence determined by the deep learning models as well as the reading center. Top row: RP240 hybrid model; middle row: RP340 hybrid model; Bottom row: the reading center. Left column: small-size EZ; middle column: medium-size EZ; right column: large-size EZ.

Figure 5 illustrates three examples of EZ area presence determined by the DL models as well as by the reading center. The top row was a single measurement of EZ area by a RP240 hybrid model; the middle row by a RP340 hybrid model; and the bottom row by the reading center. The left column showed a case of small-size EZ; the middle column a case of medium-size EZ; and the right column a case of large-size EZ.

The first example on the left column in Figure 5 was a case where the RP240 hybrid model overestimated small-size EZ area by 14.7% when compared to the reading center, while the EZ area measured by the RP340 hybrid model was closer to that of the reading center (4.2% smaller when compared to that of manual grading), suggesting that after trained on the new RP340 dataset, the RP340 hybrid model may perform better in segmentation of small-size EZ when compared to the RP240 hybrid model.

The second example in the middle column of Figure 5 was a case of medium-size EZ area estimation where both RP240 and RP340 models underestimated the EZ area when compared to the manual grading. However, the difference between the RP340 hybrid model and the manual grading was smaller than that for the RP240 hybrid model (17 vs. 30% smaller, respectively), demonstrating the impact of the extra B-scan images added to the training dataset on the model’s performance in medium-size EZ area measurement.

The third example on the right column in Figure 5 also showed a case where the large-size EZ area measured by the RP240 hybrid model was smaller than that by the RP340 model, with the result of the RP340 model much closer to that of the reading center. The RP240 hybrid model underestimated the EZ area by 15% when compared to the manual grading, while the difference of the estimated EZ areas between the RP340 hybrid model and the reading center was only 1.2%. In addition, this example illustrated that, while percent difference vs. the manual grading seems comparable for the measurements of different size EZ, the absolute difference is larger for large-size EZ measurements as shown by the error bars in Figure 3. As a matter of fact, the other two out of three measurements by each model for this case showed that EZ area was either closer or larger than that determined by the manual grading.

## Discussion

The results of this study demonstrate that automatic EZ area measurements generated from our DL models were in excellent agreement with those by the manual grading of the reading center, with a correlation coefficient >0.99 for both U-Net model and hybrid models as well as with a mean difference ± SD of -0.137 ± 1.131 mm^2^ and -0.082 ± 0.825 mm^2^ for the original (RP240) and the improved (RP340) hybrid model, respectively. Our findings are consistent with a recent study by Loo et al. (26) showing a close agreement of EZ area estimates between a deep learning-based algorithm and experienced human graders (a mean DSC ± SD of 0.79 ± 0.27, a mean absolute different ± SD of 0.62 ± 1.41 mm^2^ with a correlation of 0.97). The similarity between the performances of deep learning models and the manual grading for EZ area measurements suggests that deep learning may provide effective tools to significantly reduce the burden of reading centers to analyze OCT scan images in RP. In addition to EZ area, our deep learning models can also generate the measurements of photoreceptor outer segment volume and thickness to provide additional retinal layer metrics to facilitate the study of structure and function relationship (37) and to assess disease progression and future treatment trials in RP.

The pixel-wise comparison of the similarity between the EZ area segmentation by the DL models and EZ area annotation by the manual grading of the reading center also showed the excellent agreement between the DL models and the human graders for EZ area larger than 1 mm^2^. For EZ area smaller than 1 mm^2^, dice coefficient reduced with the decrease of EZ area. On the other hand, Bland-Altman plots showed that the size of EZ area measured by the DL models was in close agreement with that of the manual grading when EZ area was less than 1 mm^2^, suggesting that lateral shift of the EZ area determined by the DL models relative to that by the manual grading was the main cause of the reduced dice coefficient for very small-size EZ, as predicted by the fixed shift model shown in Figure 1 where a fixed shift of 50 pixels (0.315 mm) generated an output closely fit to the data of the dice coefficients. It is worth to point out that the mean difference of EZ width measured by the DL model and by the manual grading was around 0.2 to 0.3 mm as we reported previously (1, 29).

The results of this study also provide evidence to support our hypothesis that training data imbalance or under representation with the increase of EZ areas in our original U-Net and hybrid models (1) may be one of the reasons for the underestimation of the width or area for the medium-size EZ where EZ transition zone is approaching to or beyond the edge of the macula. Same as what we observed in our previous studies with internal evaluation datasets (1, 28), the original RP240 models underestimated the area of medium-size EZ in the external evaluation dataset employed in this study. By increasing the number of OCT B-scan images with EZ transition zone extended to and beyond the macula in the training dataset, we have shown in this study that the difference of medium-size EZ area measurement was reduced between the new RP340 U-Net and the manual grading when compared to the original RP240 U-Net, resulting in the improvement of linear regression slope as demonstrated in Figure 3 and Table 1. Although the overall percent cases of medium- to large-size EZ was increased only by about 7% from 49% in the original RP240 training dataset to 56% in the RP340 training dataset, the new cases added may provide significant amount of information not presented or may enhance the weak information presented in the original training dataset for the medium–size EZ. In addition, the increased cases of small-size EZ in RP340 resulted in the improvement of the model’s performance as shown in Figure 4. The increased cases of small- to medium-size EZ in the training dataset also improved dice similarity coefficient for EZ area larger than 1 mm^2^.

To handle segmentation errors by U-Net, we have proposed a hybrid model that combines U-Net for fast semantic segmentation and a sliding-window (SW) CNN model for refinement (1). Our previous study demonstrated that, by correcting misclassification of retinal layers from U-Net, the hybrid model improves automatic segmentation of retinal layer boundary lines from OCT scan images in RP, with inner limiting membrane (ILM) benefited the most (1) and EZ width estimation not much affected. The results of this study were consistent with our previous findings that EZ area measurement by the hybrid model was comparable to that by the U-Net. While the correction of the segmentation errors of the photoreceptor outer segment layer for small-size EZ generated from U-Net was one of the objectives of the hybrid model design, where isolated small pieces of EZ, including small EZ’s at the center of a scan, were re-examined using the SW model for confirmation or elimination (1). The results from this study demonstrated that such strategy may not work well in the current hybrid model for the estimation of the area of small-size EZ. For instance, the U-Net segmentation of medium- to large-size EZ band in a B-scan image was not re-examined in the hybrid model by the SW model using the same rules applied to other layer boundary lines (ILM, INL, RPE, and BM) to check for the breaks or gaps along an EZ band, considering that more complex rules may be needed to examine multiple disconnected local EZ bands to determine which is legit and which is segment error. In addition, we observed that U-Net tends to make more segmentation error at the ends (or tails) of an EZ band, and such potential errors were not checked by the current hybrid model, which may contribute to the burred edges observed in the examples of Figure 5. Future improvement of the hybrid model is needed to reduce segmentation noise of EZ bands, especially around the EZ transition zone, while preserving the segmentation of the actual EZ boundary line, so that the accuracy of the hybrid model for EZ band segmentation can be improved further.

There were other limitations of the study. While our results (Figure 4) showed that the models trained on a new dataset including more B-scan images with extended EZ sizes improved the agreement with the manual grading for the area estimation of small- to medium-size EZ, it appears that there was some residual underestimation for medium-size EZ, which may suggest that more OCT B-scan images with medium-size EZ could be added to the training dataset to potentially reduce the underestimation of medium-size EZ areas further. In addition, only mid-line B-scan images were used for the model training so far. While the trained models work well as demonstrated in this study, including off-center B-scan images from volume scans in training dataset may further improve the model’s performance. On the other hand, the external evaluation dataset had a much smaller number of cases of large-size EZ (≥30 mm^2^). Thus, the results of this study did not provide sufficient evidence to determine the impact (improvement, no change, or deterioration) of the re-trained model on the area estimation for large size EZ. More evaluation data are needed to assess the performance of the DL models for automatic measurement of large EZ areas. As an additional testing, we applied the re-trained RP340 model to our internal testing dataset of 160 mid-line B-scans employed in our previous studies (1, 29). In this internal testing dataset, 17 B-scans had EZ width ≤ 1.0 mm (mean ± SD = 0.78 ± 0.19 mm); 72 had EZ width > 1 mm and ≤ 3.0 mm (1.96 ± 0.60 mm); 48 had EZ width > 3 mm and ≤6 mm (4.12 ± 0.85 mm); and 23 had EZ width > 6 mm (7.27 ± 0.98 mm). The preliminary analysis confirmed the findings reported in the Results. When compared to the RP240 model, the RP340 model showed a trend of improved correlation with the manual grading for the measurement of EZ width (correlation coefficient changed to 0.988 from 0.981), increased linear regression slope (changed to 0.951 from 0.930), and reduced CoR (changed to 0.582 mm from 0.728 mm). Mean differences of EZ width ± SD between the models and the manual grading were 0.013 ± 0.297 mm and -0.055 ± 0.373 mm for RP340 and RP240 models, respectively.

In addition to the small number of cases of medium- to large-size EZ in the external testing dataset employed in this study, the residual insufficient number of B-scan images with medium- to large-size EZ bands in the training dataset may be a contributing factor to the larger error bars (larger absolution difference) for the mean EZ area measurements by the same model but trained at different time on the same training dataset (Figure 3). It needs to be determined whether the standard deviation of mean area measurement for large-size EZ can be reduced with more B-scans having extended EZ added to the training dataset, which could provide a guideline for how many measurements are needed to obtain a reliable estimate of an EZ area. Finally, the current study only included the cross-sectional data of the baseline of RUSH2A study. Longitudinal studies are needed to evaluate the power of deep learning models to detect disease condition change in RP. Once the longitudinal data of RUSH2A study is available, we will conduct the analysis to assess the performance of deep learning models comparing to human graders to monitor disease progression in RP.

## Data Availability Statement

The raw data supporting the conclusions of this article will be made available by the authors, without undue reservation.

## Ethics Statement

The studies involving human participants were reviewed and approved by IRB of UT Southwestern Medical Center. Written informed consent to participate in this study was provided by the participants’ legal guardian/next of kin.

## Author Contributions

Y-ZW was responsible for overall design of the study, the development of the deep learning models, data analyses, and manuscript writing. DB provided the datasets for the training of deep learning models and contributed to the design of the study, data analysis, and manuscript writing. Both authors contributed to the article and approved the submitted version.

## Author Disclaimer

The source of the RUSH2A data is the Foundation Fighting Blindness Consortium, but the analyses, content, and conclusions presented herein are solely the responsibility of the authors and may not reflect the views of the Foundation Fighting Blindness.

## Conflict of Interest

The authors declare that the research was conducted in the absence of any commercial or financial relationships that could be construed as a potential conflict of interest.

## Publisher’s Note

All claims expressed in this article are solely those of the authors and do not necessarily represent those of their affiliated organizations, or those of the publisher, the editors and the reviewers. Any product that may be evaluated in this article, or claim that may be made by its manufacturer, is not guaranteed or endorsed by the publisher.

## Footnotes

1 Patients from the European sites of the RUSH2A study are not permitted to share data for secondary research purposes since GDPR consents were not signed.
